# The role of TRPV1 in RA pathogenesis: worthy of attention

**DOI:** 10.3389/fimmu.2023.1232013

**Published:** 2023-09-08

**Authors:** Yuan Qu, Yang Fu, Yuan Liu, Chuanguo Liu, Bing Xu, Qian Zhang, Ping Jiang

**Affiliations:** ^1^ First College of Clinical Medicine, Shandong University of Traditional Chinese Medicine, Jinan, China; ^2^ Institute of Chinese Orthopedics and Traumatology, Shandong Wendeng Osteopathic Hospital, Weihai, China; ^3^ Experimental Center, Shandong University of Traditional Chinese Medicine, Jinan, China; ^4^ Department of Rheumatology, Affiliated Hospital of Shandong University of Traditional Chinese Medicine, Jinan, China; ^5^ Science and Technology Department, Affiliated Hospital of Shandong University of Traditional Chinese Medicine, Jinan, China

**Keywords:** TRPV1, rheumatoid arthritis, pathogenesis, pain, inflammation, treatment, ion channels

## Abstract

Transient receptor potential cation channel subfamily V member 1 (TRPV1) is a Ca^2+^permeable, non-selective cation channel that is found primarily in sensory nerve fibres. Previous studies focused on pain transmission. However, recent studies have found that the TRPV1 channel, in addition to being associated with pain, also plays a role in immune regulation and their dysregulation frequently affects the development of rheumatoid arthritis (RA). A thorough understanding of the mechanism will facilitate the design of new TRPV1-targeted drugs and improve the clinical efficacy of RA. Here, we provide an updated and comprehensive overview of how the TRPV1 channel intrinsically regulates neuronal and immune cells, and how alterations in the TRPV1 channel in synoviocytes or chondrocytes extrinsically affect angiogenesis and bone destruction. Rapid progress has been made in research targeting TRPV1 for the treatment of inflammatory arthritis, but there is still much-uncharted territory regarding the therapeutic role of RA. We present a strategy for targeting the TRPV1 channel in RA therapy, summarising the difficulties and promising advances in current research, with the aim of better understanding the role of the TRPV1 channel in RA pathology, which could accelerate the development of TRPV1-targeted modulators for the design and development of more effective RA therapies.

## Graphical Abstract

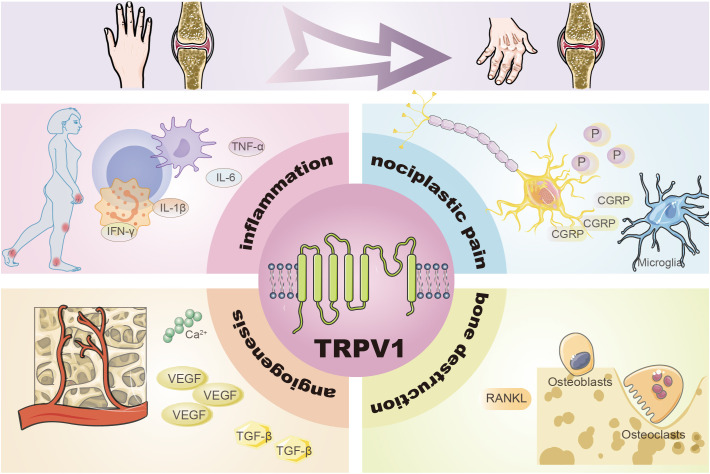

## 1 Introduction

Rheumatoid arthritis (RA) is a chronic autoimmune disease with morning stiffness, swelling, pain, and functional impairment of the joints as the main clinical manifestations (1). The pathological process of RA involves immune cell infiltration, excessive cytokine production, angiogenesis, and cartilage destruction, which can lead to joint ankylosis, destruction, and deformity, resulting in disability and affecting the patient’s quality of life (2).

Inflammation and pain are prominent problems in the treatment of RA. Glucocorticoids, non-steroidal anti-inflammatory drugs (NSAIDs), and disease-modifying anti-rheumatic drugs (DMARDs) are currently available to improve the patient’s condition, and bio DMARDs or targeted synthetic DMARDs can be used when improvement is not evident (1, 3, 4). Despite the many drugs currently available, the response to treatment remains unsatisfactory, usually between 50% and 70%, and a significant number of patients have poor treatment outcomes (5).In clinical practice, if arthritis does not respond to initial treatment, we should change the treatment strategy as soon as possible. Some DMARDs suppress the immune system, resulting in an added risk of infection in RA patients and further aggravating the disease condition (6–8). Therefore, there is a real requirement to develop therapeutic agents for new targets to improve the outcome of RA treatment and prognosis.

TRPV1 is the first identified member of the vanilloid receptor subfamily of the TRP ion channel.TRPV1 is not only involved in the sensation of heat and pain but is also associated with abnormal immune cell function and the production of inflammation in the body, among other mechanisms (9). It was revealed that synovial fibroblasts from RA patients express TRPV1 (10). Compared to TRPV1^+/+^ animals, TRPV1^-/-^ animals exhibited reduced pain and reduced joint inflammation following complete Freund’s adjuvant (CFA)-mediated induction of arthritis (11). Injection of TRPV1 antagonists A-889425 and JNJ-17203212 systemically reduces pain behaviour and decreases peripheral A and C fibre joint afferent nerve and injury receptor firing in a model of arthritis (12, 13).In this review, we summarise the TRPV1 channel expression associated with RA reported to date, and the impact of channel alterations on the pathogenesis of RA inflammation, pain, angiogenesis, and cartilage destruction. The impact of interfering with the TRPV1 channel on the above mechanisms is also listed in detail to further assess the therapeutic potential of targeting TRPV1 in RA to guide future research efforts.

## 2 Structure and function of TRPV1

**Figure 1:**
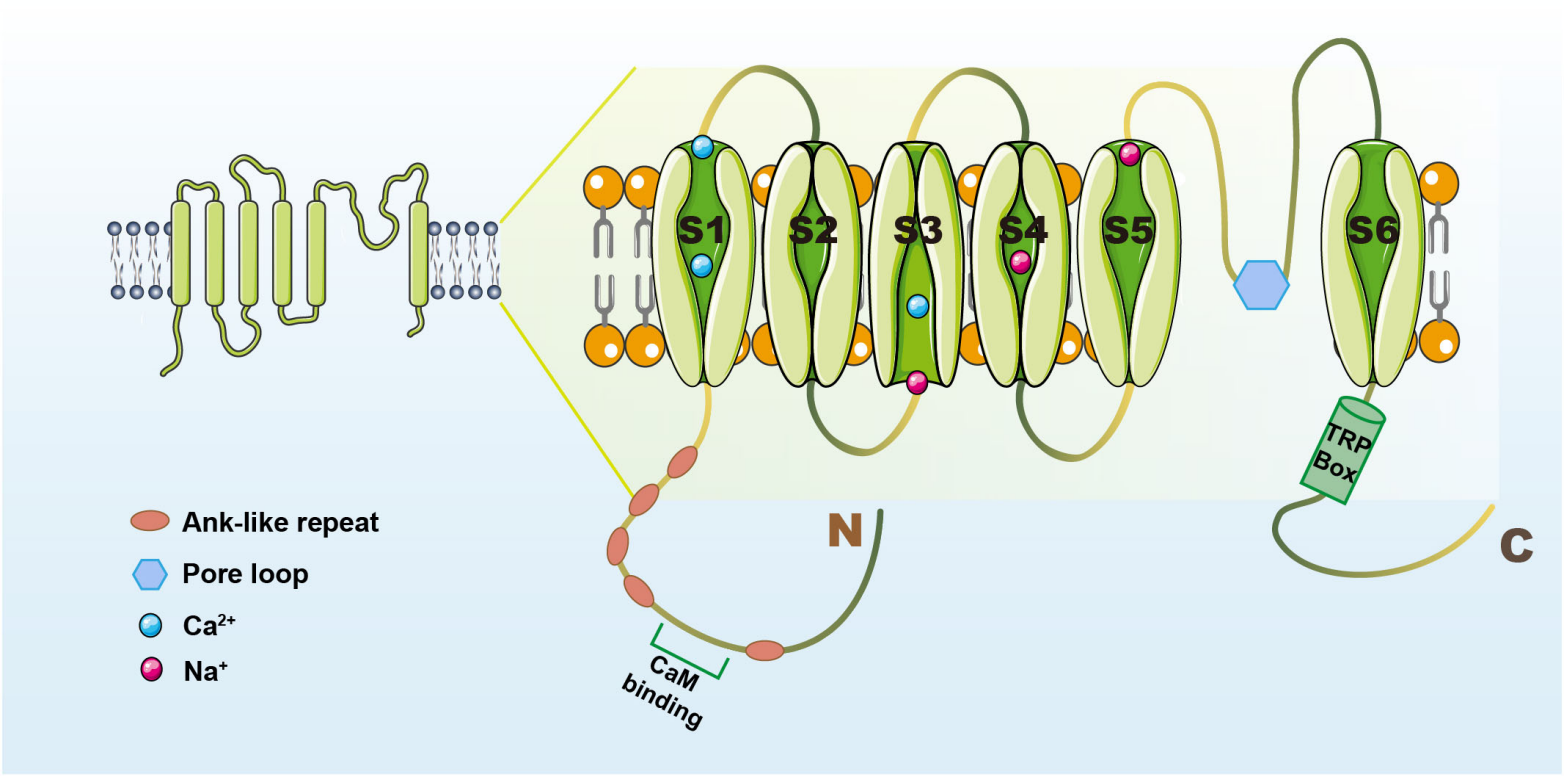
The structure of the TRPV1 channel. TRPV1 contains N- and C-termini and intermediate transmembrane structural domains (S1-S6). The N-terminus has a cam-binding site and an anchor protein repeat structural domain (16). The C-terminus has a TRP-box near the sixth transmembrane structural domain that can influence channel function (16). The pore loop region is located between S5 and S6, and when activators act on TRPV1, the structure of S5-6 is altered and contributes to Ca^2+^-based cation inward flow (15).

TRPV1 is a non-selective cation channel, which was characterized as a receptor for capsaicin (CAP) by the Julius laboratory in 1997 (14). TRPV1 has a tetrameric structure, similar in structure to most TRP channels, and consists of three parts: the N- and C-termini located in the cell, and six transmembrane regions (S1-S6), with the pore loop region located between S5 and S6 (15). TRPV1 has a long N-terminal containing an anchor protein repeat domain and a C-terminal containing a TRP-box close to the sixth transmembrane structural domain (16), the N-terminal plays a role in the sensitivity of the channel to activators, and the C-terminal TRP-box mainly affects channel stability and function. Binding sites on the repeat structural domain of TRPV1-anchored proteins can bind to nucleotide triphosphates like ATP and calmodulin at the identical site (17, 18), upon binding, these molecules modify the sensitization of TRPV1 and modulate its function (19). The structure of the TRPV1 channel is summarized in **Figure 1**.

TRPV1 is mainly expressed in sensory nerve fibres, including unmyelinated C nerve fibres and small-diameter myelinated A nerve fibres. As a result, early studies focused on pain transmission, including thermal and inflammatory pain. However, as research progressed, it was discovered that TRPV1 was not only found in neuronal cells but also in other cell types, including RA synovial fibroblasts and human immune cells such as dendritic cells(DCs), macrophages or T lymphocytes (20). TRPV1 is tightly correlated with autoimmune diseases. TRPV1 knockout (TRPV1 KO) mice confirm a key role for TRPV1 in pain and inflammation (21). Therefore, understanding its anti-inflammatory and analgesic mechanisms and how TRPV1-targeted therapies work in joints and other tissues may provide new options for the treatment of inflammation and pain in RA patients.

## 3 The potential role of TRPV1 in RA

### 3.1 TRPV1 is involved in organismal inflammation

Inflammation is a key pathological manifestation in the pathogenesis of RA, and there is increasing interest in the mechanisms involved, mainly related to the over-activation of immune cells and dysregulation of inflammatory cell secretion (22–25). TRPV1 has been reported to show increased expression at the mRNA and protein levels in synovial fibroblasts from RA patients, correlating with RA inflammation and pain (10). The use of TRPV1 antagonists or knockouts relieves joint inflammation (26), again confirming its key role. Further studies should focus on the cell types expressing TRPV1 and the regulatory role of TRPV1 on cells, in relation to cytokine release, to elucidate the true function of TRPV1 in the pro-inflammatory process.

#### 3.1.1 Immune cell infiltration

The site of RA is mainly confined to synovial joints and is closely associated with immune cell infiltration (27). TRPV1 was identified as present in cells of either the innate or adaptive immune system, regulating immune cell activation and influencing their function (28–32). TRPV1 is involved in Ca^2+^ signalling and the transduction of external stimuli (e.g. temperature or pH). Ca^2+^ is a well-known second messenger and plays a pivotal role in the activation of immune cells, proliferation, cytokine secretion and other functions (33, 34). Therefore, TRPV1 appears to be an essential participant in the regulation of immune cells.

##### 3.1.1.1 T lymphocytes

The immunopathogenesis of RA spans decades, with T-cell dysregulation found in the asymptomatic autoimmune abnormal phase of RA (a period of autoantibody production to post-translational modified proteins), the acute synovitis phase, and the chronic destructive synovitis phase (35). Aberrant T cell differentiation is fundamental in promoting the remodelling of the immune system prior to the disease (36). Another way in which pathogenic T cells drive chronic inflammation is associated with the formation of organised lymphoid structures (37), which provide strength and persistence to inflammatory immunity. Moreover, synovial T cells are an important source of inflammatory factors such as IFN-γ (38), IL-17 (39), and TNF-α (40), and are a key bridge between cytokines and adaptive immune abnormalities and tissue remodelling.

Expression of TRPV1 was detected in human primary T cells, Jurkat T cell line, and mouse spleen T cells (29, 41–43). TRPV1 is positioned on the T cell plasma membrane and is one of the pivotal components of the T cell receptor (TCR) signalling cascade. TRPV1 activation and function can be regulated through TCR-induced signalling pathways (44). TRPV1 is rapidly recruited to the TCR cluster in an Src-dependent manner after TCR stimulation (42), which is an important way to increase Ca^2+^ concentration in CD4^+^ T cells. Resiniferatoxin, a specific agonist of TRPV1, leads to increased Ca^2+^ influx in T cells (29). Elevated intracellular Ca^2+^ is imperative for promoting T cell functions, such as activation, proliferation, differentiation and exerting effector functions (45). Genetic deletion or pharmacological inhibition of TRPV1 can attenuate the pro-inflammatory phenotype of CD4^+^ T cells (42, 46). Knockdown of TRPV1 in human primary CD4^+^ T cells reduces the expression of CD25 and shared epitope-positive HLA-DR alleles and diminishes the generation of anti-citrullinated protein antibodies (ACPA) in RA patients (42).In addition, TRPV1 antagonists can reduce the production of inflammatory factors. Compared to wild-type(WT), TRPV1^-/-^CD4^+^ T cells secreted fewer cytokines (IFN-γ, IL-17A, IL-2, IL-10, IL-4 and TNF) following anti-CD3 + 28 stimulation, and their reduced cytokine production may be due to a decrease in TCR-induced the TRPV1 channel-mediated Ca^2+^ influx (42). Although the role of TRPV1 and T cell activation in the pathogenesis of RA remains to be clarified, the role of TRPV1-mediated Ca^2+^ influx in T cell proliferation and activation is undisputed. Considering the important pathological role of T-cell activation in RA, it can be inferred that targeting and regulating TRPV1 to restore normal physiological functions of T-cells holds great promise in the treatment of RA.

##### 3.1.1.2 Macrophages

TRPV1 has been reported to be involved in macrophage-related immune mechanisms (47). Abnormal activation of pro-inflammatory macrophages in synovial tissue is detected in early RA (48, 49). When pro-inflammatory macrophage M1 are over-polarized, the secretion of cytokines such as IL-1β, IL-6 and TNF-α increases *in vivo (* 48, 50), inducing inflammation production and leading to high disease activity in RA. TRPV1 hyperactivation promotes M1-type pro-inflammatory macrophage activation by altering macrophage status (51). An imbalance in the M1/M2 ratio of articular synovial macrophages is a distinctive feature of the acute inflammatory phase of RA (52, 53). Not only are macrophages themselves a source of inflammation, but they are also associated with the activation of T cells. Macrophages can present non-self antigens to nascent T cells, by releasing cytokines and growth factors that elicit Th1 or Th2-mediated immune responses and modulate inflammation *in vivo* (54). Pretreatment of mouse macrophages with TRPV1 inhibitor AMG9810 or CPZ significantly inhibited the expression of pro-inflammatory cytokines IL-6, IL-1β and IL-18, as well as cyclooxygenase 2 (COX-2) (55). An opposite result has been shown, with TRPV1 agonist treatment significantly reducing macrophage polarisation, attenuating synovial inflammation and minimising cartilage destruction and bone formation (31).

Macrophage polarization derangement is a key mechanism of bone destruction in RA. The degree of synovial macrophage infiltration is closely related to the degree of joint erosion (49). An interesting subpopulation of macrophages called arthritis-associated osteoclast macrophages, has been discovered in the synovial fluid and tissues of RA patients (56). These macrophages are characterised by a high osteoclastogenic potential (56). TRPV1 activation facilitates the mobilization of M1-type pro-inflammatory macrophages. Inflammatory macrophages are involved in joint surface erosion through the production and release of matrix metalloproteinases (57). M1-type macrophages are intimately associated with osteoblasts. During the erosive phase of the disease, chemokine CCL21 enhances RA osteoclastogenesis by driving Th17 polarization through M1-type macrophages. Thus, CCL21-mediated differentiation of M1-type macrophages linked to Th17 cells extends joint inflammation into bone erosion (57). The macrophage-osteoclast axis may be an important mechanism of bone destruction in RA (58). TRPV1 can reduce inflammation and osteoclast production by regulating the M1/M2 macrophage imbalance and is expected to be a new therapeutic target for RA.

TRPV1 and macrophages are not limited to their involvement in inflammation and bone destruction but are also associated with mechanical pain and burning sensations. IL-23/IL-17A/TRPV1 axis activation contributes to macrophage-sensory neuron crosstalk, creating mechanical pain (59). Radiofrequency irradiation lowers TRPV1 activation, diminishes neuropeptide expression, attenuates neuropeptide-induced macrophage activation, and ultimately reduces inflammatory factor expression and burning pain *in vitro* models (60). The mechanism by which TRPV1 is involved in macrophages to regulate inflammation and bone destruction in the body has been well clarified. Yet, the mechanism of TRPV1-induced neuropeptide expression and macrophage activation in RA patients with burning joint pain remains to be further elucidated.

##### 3.1.1.3 Dendritic cells

TRPV1 has been proven to be expressed in mouse and human DCs at the protein and gene levels (28, 61, 62). The TRPV1 agonist CAP exhibits a dose-dependent effect of inducing DCs differentiation and prompting DCs activation. TRPV1 binds to immature DCs and promotes DCs maturation through antigen presentation and upregulation of co-stimulatory molecules (61). Substantial amounts of DCs are recruited in the synovial fluid and tissues of RA patients and are integral in the pathogenesis of RA (63, 64). DCs specialized antigen-presenting cells bridge innate and adaptive immunity (65), drive Th1/Th2 imbalance, activate B cells and follicular helper T cells (Tfh) (66, 67), and stimulate the body to generate high levels of autoantibodies, resulting in an inflammatory response.

TRPV1 regulates DCs function, and sustained opening of the TRPV1 channel promotes activation of calcium-regulated neuro phosphatase/NFATc2 signalling in DCs, impairing Ca^2+^ homeostasis in DCs, enhancing Th17 cell differentiation, inducing cytokine secretion and strengthening susceptibility to inflammatory factors (68). Excessive secretion of chemokines by synovial DCs in RA patients and recruitment of immune cells with pro-inflammatory functions, such as macrophages and neutrophils (69, 70). Overexpression of NF-κB was found in RA synovial DCs, causing upregulation of nuclear RelB, a binding protein for NF-κB, and promoting inflammation. Similar manifestations were found in inflammatory bowel disease (IBD) mice, where activation of TRPV1 promoted DCs recruitment and activation compared to TRPV1^-/-^, exacerbating the colitis manifestations of the model (68).

However, studies have suggested the opposite, that the failure of CAP to induce changes in intracellular Ca^2+^ or membrane currents in DCs does not support the expression of the TRPV1 channel in DCs (71). The reason for this may be that although CAP can agonize the TRPV1 channel, numerous studies have shown that TRPV1 receptors may not be the only target of CAP. The specific role of TRPV1 in the regulation of DCs by CAP remains unclear. Although several studies have supported the idea that TRPV1 regulates DCs and influences the inflammatory response of the body. Further validation at the cellular level using TRPV1^-/-^ or agonists and inhibitors to elucidate the mechanisms of TRPV1 regulation of inflammation in DCs and RA patients is an important next step in the research.

#### 3.1.2 Cytokine production

##### 3.1.2.1 IL-1

Overproduction of IL-1β causes vasodilation, promotes granulocyte recruitment to inflamed tissues and induces prostaglandin (PG) expression, contributing to acute joint inflammation and pain (72, 73). IL-1β also stimulates osteoclast formation through the induction of osteoclast genesis in regulatory T-cells (Tregs), contributing to bone erosion and joint function reduction (74, 75). TRPV1 is an integral pathway for IL-1β release. Studies have shown that injurious TRPV1^+^ axons co-express IL-1R1 and are tightly correlated with infiltrating IL-1β^+^ cells. In both mouse and human models, IL-1R1 was found to be highly expressed through a subpopulation of TRPV1^+^ dorsal root ganglion neurons (76). TRPV1 channel induces Ca^2+^ influx in the extracellular medium and increases cytosolic Ca^2+^ concentration, possibly through increased nuclear NF-κB phosphorylation leading to increased IL-1β release (77). Pretreatment with TRPV1 antagonist significantly eliminates IL-1β-induced pain (78). No elevation of IL-1β was seen in the TRPV1 KO mouse model compared to WT mice (79, 80). Inhibition of IL-1 secretion through modulation of the TRPV1 channel to attenuate joint inflammation and pain merits further exploration.

##### 3.1.2.2 IL-6

Elevated levels of synovial TRPV1 in RA patients are accompanied by abnormal secretion of IL-6 (81). TRPV1-induced IL-6 secretion plays an instrumental role in patients’ pain, inflammation, and joint destruction. Activation of TRPV1 operates as an inducer of inflammatory signalling and collaborates with neuropeptides to augment the production of the cytokine IL-6. However, this synergistic mechanism does not affect healthy synovial cells, and only inflammation-initiated cells can generate IL-6 through the activation of the TRPV1 channel (81). IL-6 plays a checkpoint role in the differentiation pathway of naive T cells to pro-inflammatory Th17 cells or Tregs (82). IL-6 acts as a chemoreceptor for monocytes at the site of inflammation, affecting signalling molecules such as Toll-like receptors (TLR) and promoting angiogenesis (83). IL-6 mediates the induction of bone resorption by TNF-α and IL-1. IL-6 stimulates the release of osteoblast nuclear factor-κB receptor activator (RANK) ligand (RANKL) and destroys bone via the RANK/RANKL/osteoprotegerin (OPG) pathway (84). The simultaneous destruction of the vascular opacification leads to irreversible damage to the joint (85). The use of TRPV1 inhibitors reduces the body’s IL-6 levels (86). TRPV1 KO mice secreted less IL-6 compared to WT mice (87). Thus, TRPV1-induced IL-6 secretion has an essential role in inflammatory, neurogenic and pressure pain in the body.

##### 3.1.2.3 IL-8

TRPV1 has a regulatory effect on IL-8. It was revealed that activation of TRPV1 often induces IL-8 formation. Elevated levels of TRPV1 in the hyperosmotic state and response to dramatic temperature changes stimulate MAPK and NF-κB activation and mediate an increase in the chemokine IL-8 (88). IL-8 is a key chemokine that promotes neutrophil migration (89). IL-8 activates neutrophils, stimulates neutrophil extracellular trap formation, promotes their degranulation, produces respiratory bursts, releases superoxide and lysosomal enzymes, and facilitates the activation and recruitment of neutrophils at sites of inflammation (90). IL-8 also has the capability to appeal to and activate monocytes, and large quantities of inflammatory cells infiltrate the interstitial matrix of articular cartilage and bone (91, 92). IL-8 can also stimulate angiogenesis, resulting in the formation of RA vascular opacities (91). The use of the TRPV1 agonist CAP confirms the regulatory function of TRPV1 on IL-8 (93). Reduced local pH is an essential pathological feature of RA. Acidic conditions can contribute to elevated IL-8 mRNA expression (94). Intriguingly, the selective TRPV1 antagonist 5’-iodoresiniferatoxin reduced the production of IL-8 under acidic conditions and attenuated the inflammatory state of the organism (95).

IL-8 also has osteoclastogenic activity. IL-8 promotes the formation of pro-osteoblast-like cells (OCL) in an environment with anti-RANKL antibodies, demonstrating that IL-8 may compensate for RANKL function during the induction of OCL in a low-RANKL environment and accelerate the process of RA bone destruction (96). TRPV1 may have an impact on bone destruction in RA patients by mediating IL-8 secretion.

##### 3.1.2.4 IL-17

It was revealed that TRPV1^+^ injury receptors are closely related to the production of IL-17. IL-17 can induce aggregation of neutrophils and monocytes in the circulatory system, activates a series of inflammatory cascades and plays a fundamental role in RA (97). TRPV1 activates the IL-23/IL-17 axis, which mediates inflammation through macrophage-neuron crosstalk drive (98). TRPV1 KO abrogates IL-23/IL-17 axis-induced inflammation. At the same time, IL-17, in turn, acts on TRPV1, the two interact through neural-immune interactions, contributing to the body’s mechanical pain (59). The use of TRPV1 agonists CAP, SA13353 [1-[2-(1-adamantyl)ethyl]-1-pentyl-3-[3-(4-pyridyl)propyl]urea] also demonstrated anti-inflammatory effects. CAP administration inhibited the expression of the IL-23/IL-17 pathway in psoriasis models, alleviated the microscopic appearance of lesions and reduced the secretion of various cytokines (99). SA13353 suppresses the recruitment and production of IL-17-producing cells and decreases inflammation in the body (100). TRPV1 KO confirmed its induction of IL-17 production causing inflammation in the body, but its agonist use also reduced inflammation in the body, suggesting that both agonists and inhibitors of TRPV1 can exert anti-inflammatory effects via IL-17 secretion, and the mechanism of action requires further exploration.

##### 3.1.2.5 TNF-α

TNF-α induces inflammation and promotes osteoclastogenesis, which is critical in the pathogenesis of RA. TNF-α inhibitors are widely used to treat RA and can mitigate RA symptoms by preventing the pro-inflammatory signalling pathway mediated by TNF receptor 1 (TNFR1) (101). TRPV1 promotes TNF-α production and is also induced by TNF-α, both of which exert synergistic inflammatory effects. Pretreatment of isolated rat vagus nerves with TNF-α enhances the response of sensory neurons to TRPV1 agonists and mediates increased TRPV1 expression and Ca^2+^ influx (102). In TNF-α overexpressing mice, elevated TRPV1 levels and elevated Ca^2+^ influx levels were demonstrated, indicating that TNF-α overexpression induced TRPV1 sensitization (103). TNF-α-triggered thermal and mechanical hypersensitivity in the body is mediated by TRPV1 signalling downstream of TNFR1 receptor activation, respectively, and sensitization of injury receptors is dependent on TNFR1 expression (104).

Also, TRPV1 mediates the production of TNF-α. Paeoniflorin inhibits TRPV1 expression in foot pad tissue samples, suppresses inflammatory cytokine TNF-α production and ameliorates inflammation and pain in mice in an LPS-induced acute pain model (105). However, the analgesic effect of paeoniflorin can be significantly reversed by CAP, suggesting that TRPV1 channel activation mediates the release of inflammatory factors such as TNF-α, leading to inflammatory pain.

TRPV1 and TNF-α exhibit a close reciprocal relationship and their mutual regulatory role cannot be ignored. Of interest is that in two studies, TRPV1 agonists showed a dual anti-inflammatory and pro-inflammatory effect and inflammatory cytokines such as TNF-α also showed a paradoxical elevation and inhibition (106, 107). The mechanism of TRPV1’s role in this requires further clarification. It is undeniable that TRPV1 is tightly associated with TNF-α, and blocking the reciprocal relationship may reduce the inflammatory response in RA patients, and may also delay bone destruction, diminish disability and improve the quality of patient survival.

##### 3.1.2.6 IFN-γ

Overproduction of IFN-γ in RA patients drives the recruitment of synovial neutrophils, which is accompanied by high disease activity and leads to persistent inflammation (108, 109). TRPV1 mediates IFN-γ production. In a model of inflammation induction, both inflammatory genes IFN-γ and TRPV1 channel were upregulated, and TRPV1 agonists systematically increased T cell counts, enhanced CD8^+^ T cell recruitment and induced overproduction of IFN-γ in healthy mice (110, 111). Nevertheless, a novel TRPV1 agonist, SA13353, attenuated IFN-γ cytokine levels, possibly associated with the desensitization of TRPV1 (100). Although TRPV1 channel opening correlates with IFN-γ secretion, there is still a lack of strong evidence to support this. Whether TRPV1 KO affects the secretion of IFN-γ and thus the development of RA still needs to be further explored.

Activation of the TRPV1 channel is known to cause inflammation *in vivo*, but there is growing evidence that it has anti-inflammatory effects. TRPV1 is expressed in a variety of immune cell subpopulations, and activation of the channel is closely synergistic with the release of regulatory proteins, and inflammatory factors. These synergistic relationships may influence the final outcome of TRPV1 activation.

### 3.2 TRPV1 regulates nociplastic pain

Pain is a distinctive feature of RA and a major source of poor prognosis and low quality of life for patients. As pain caused by RA is traditionally considered to be a direct result of peripheral inflammation, doctors have traditionally considered pain to be a marker of inflammation. However, despite the success of DMARDs in suppressing inflammation, many people with RA still have pain. Pain in inflammatory arthritis has a variety of causes; peripheral inflammatory triggers, structural damage, psychosocial factors, etc. These factors are entwined with the central mechanisms of pain (112). Pain management in RA is an increasing challenge for rheumatologists. Comprehending the underlying biological mechanisms of pain is essential to improving treatment, disease management and patient health. The pathogenesis of pain can be divided into 3 categories (113): 1) Nociceptive pain, which is the response of the somatosensory system to an injurious stimulus; 2) Neuropathic pain, defined as a consequence of direct neurological damage; 3) Nociplastic pain, defined as a dysfunctional neurological response to pain management in the absence of peripheral tissue injury, somatosensory system damage or injury receptor engagement, is manifested as neuro sensitisation. Cutting-edge research confirms nociplastic pain as an essential cause of non-inflammatory pain in RA (113).

Excessive release of neuropeptides (including substance P (SP) and calcitonin gene-related peptide (CGRP) and microglia dysfunction were revealed to be crucial mechanisms in the collagen-induced arthritis (CIA) model (114). There is substantial evidence that TRPV1 is widely expressed in the CNS and that TRPV1 agonist-endocannabinoids can be used to treat pain caused by nerve sensitization. TRPV1 affects neuropeptide secretion, contributes to microglia activation and is closely associated with neuron sensitization, which may play an essential role in non-inflammatory pain in RA (115). Therefore, starting with TRPV1 and inhibiting abnormal activation of neuropeptides and microglia overproliferation may be a potential mechanism to inhibit central and peripheral sensitization and treat non-inflammatory pain in RA.

#### 3.2.1 TRPV1 affects neuropeptide secretion

TRPV1 mediates CGRP release. Immunohistochemistry showed that TRPV1 receptors and CGRP co-localized in a considerable proportion of neurons (116). Decreased calcium-regulated neuro phosphatase activity in sensory neurons leads to activation of the TRPV1 channel and increased intracellular Ca^2+^ concentration, which can cause increased CGRP secretion. Drugs reduce the pain-induced phosphorylation state of the TRPV1 channel by enhancing calcium-regulated neuro phosphatase activity, diminishing Ca^2+^ inward flow, and mediating neuro calmodulin-dependent desensitization of TRPV1 in sensory neurons, reducing subsequent CGRP neuropeptide transmitter release (117). TRPV1 KO or pretreatment with GCRP receptor antagonists significantly reduced the mechanosensitization induced by C5a, a component of the complement system, confirming that TRPV1 and CGRP receptors are key steps in the mechanosensitization process (118). Overexpression of αCGRP found in RA patients (119). Elevated TRPV1 mRNA and increased Ca^2+^ influx with the subsequent increased neuronal release of CGRP were detected in the CFA-induced pain model compared to the control group (120). As mechanical hypersensitivity develops in the disease, innervated neurons exhibit enhanced CGRP expression, as well as stronger pain manifestations (121).

TRPV1 is not just essential for promoting the synthesis and release of CGRP (122), but SP is also regulated by TRPV1. SP activation was observed in cultured RA synovial cells to stimulate the release of PGE2 and collagenase from synovial cells and promote synovial cell proliferation (123). Sensory neurons of TRPV1^+^ release SP upon stimulation in an allergic mouse model (124). In a chronic compression injury model, SP rises with TRPV1 levels. Treatment with ferulic acid reduces TRPV1 levels and subsequently reduces serum SP levels, inhibits peripheral sensitization and alleviates sciatica (125). The TRPV1 antagonist capsazepine significantly attenuates TRPV1 expression and SP release (126). Consistent with this, CAP treatment activates the TRPV1 channel and dose-dependently promotes the release of neuropeptides SP and CGRP (127). Interestingly, neuropeptides interact with TRPV1, with neuropeptides, in turn, activating TRPV1 in RA synovial fibroblasts and promoting IL-6 and IL-8 production, promoting synovial peripheral inflammation (81).

TRPV1 and the neuropeptides SP and CGRP have been shown to be abnormally elevated in RA patients. Multiple pain models demonstrate that TRPV1 mediates SP and CGRP secretion. However, the effect of TRPV1 KO or antagonists on downstream SP and GCRP neuropeptide secretion, on central and peripheral sensitization in RA models remains to be further investigated. While most anti-inflammatory drugs can target the immune cellular component of neuroinflammation, they usually fail to interfere with the neuronal component (117). Interfering neuropeptides have great potential value for the treatment of non-inflammatory pain in RA.

#### 3.2.2 TRPV1 and microglia activation

Nociplastic pain is associated with microglia overactivation (128). Various humoral factors released by microglia (such as IL-10, IL-1β), contribute to synapse formation and mediate pain caused by nerve sensitization, which may be a key mechanism connecting synaptic plasticity and pain (129). Pharmacological inhibition of microglia reverses region-specific synaptic plasticity in a pain model, reaffirming the key role of microglia in nociplastic pain (130).

Notably, TRPV1 is highly expressed in microglia. Activation of the TRPV1 channel regulates microglia function and microglia influence synaptic transmission and plasticity in neurons (131). Chronic pain model studies provide ample evidence (131): (a) Pain model cortical microglia exhibit high expression of TRPV1 mRNA and protein compared to negative controls; (b) The TRPV1 agonist CAP induces the shedding of microvesicles from the surface of microglia, increases glutamatergic synaptic activity and regulates synaptic transmission within the central nervous system; (c) Microglia change both morphologically and phenotypically upon TRPV1 activation, demonstrating an activation phenotype; (d) the TRPV1 channel is highly permeable to Ca^2+^ primarily in microglia. activation of microglial TRPV1 by CAP drives up intracellular Ca^2+^ and promotes the release of mitochondrial cytochrome c, leading to increased microglial apoptosis and autophagy (132); (e) Application of TRPV1 agonist to elicit concentration-dependent migration and chemotaxis of microglia (133). Several studies have shown that microglia exhibit hyperproliferation and increased reactivity in the CIA model. Progressive increase in microglia with escalating activation and sensitization to injurious neurons in CIA joints, closely associated with pain (121, 134). Peripheral inflammatory signals can stimulate CNS-resident microglia, prompting rapid conversion to an activated phenotype that perpetuates neuroinflammation (135).

This differential TRPV1 expression pattern in chronic pain conditions places microglia TRPV1 at the centre of a new and important mechanism, providing a link between physiological and pathological states. Demonstrating a key role for TRPV1 regulating microglia in nociplastic pain. AMG9810, a TRPV1-specific inhibitor, attenuates microglia activation, effectively attenuates mechanical hypersensitivity and reduces pain (136). The use of the TRPV1 blocker SB366791 significantly inhibited microglia migration and attenuated the development of mechanically abnormal pain and nociceptive hyperalgesia, with the same results observed in TRPV1 KO mice (133).

The above studies support the vital role of abnormal microglia activation in RA nociplastic pain. Focusing on sensitization and pain caused by abnormal microglia activation, modulating the TRPV1 channel, inhibiting microglia overactivation, and repairing neural sensitization may be a promising strategy for treating nociplastic pain in RA.

### 3.3 TRPV1 mediates angiogenesis

Angiogenesis in synovial tissue is a key pathological event in the progression of RA (137). Angiogenesis recruits inflammatory cells from the circulatory system, leading to persistent synovitis and the formation of invasive vascular opacities that further lead to cartilage destruction and exacerbate the progression of RA (137). Endothelial Ca^2+^ signalling plays a crucial role in angiogenesis. Various pro-angiogenic factors, such as Vascular endothelial growth factor (VEGF) and transforming growth factor β1 (TGF-β1), are involved in regulating endothelial cell proliferation and angiogenesis by increasing intracellular Ca^2+^ concentration (138, 139). TRPV1, a multimodal cation channel that mediates Ca^2+^ influx, is an important player in vascular endothelial cell migration, proliferation and angiogenesis (140). It was found that intraperitoneal injection of the TRPV1 ligand wogonin (a TRPV1 agonist) promoted angiogenesis in WT mice (141). The same results were observed in *in vitro* experiments (142). In contrast, TRPV1 antagonists eliminated drug-induced angiogenesis (143). TRPV1 KO and siRNA-interfered animals show a significant reduction in induced angiogenesis, and VEGF and TGF-β1 expression is inhibited in TRPV1 KO mice (144). Not only that but interestingly, recent findings suggest that TRPV1 triggers angiogenic activity independently of VFGF and that blocking the TRPV1 channel has no effect on VEGF-stimulated angiogenesis or Ca^2+^ signalling *in vitro (* 142). The TRPV1 agonist CAP exhibits an inhibitory effect on angiogenesis. Retinal microvasculature in diabetic rats exhibits increased retinal neovascularization and CAP ameliorates diabetic retinopathy by activating TRPV1 (145). Another study also demonstrated that CAP inhibits VEGF-induced endothelial cell proliferation, migration and angiogenesis (146). It is worth noting that TRPV1 is not the only channel activated by CAP, and the above studies did not explore the role of the TRPV1 channel in CAP anti-angiogenesis, which may be related to other mechanisms of CAP action *in vivo*. The role of TRPV1 in angiogenesis has been repeatedly demonstrated through technical means such as gene knockout, interference and inhibitors. Thus, modulation of the TRPV1 channel may be a possible means of inhibiting RA angiogenesis.

### 3.4 Potential role of TRPV1 in joint destruction

Cartilage destruction in the affected joints is the main pathological feature of RA that causes disability. Cartilage destruction occurs rapidly from the onset of RA and can lead to joint deformation and functional deterioration. Therefore, controlling cartilage destruction is an important part of treatment to reduce the disability rate of RA (147). Abnormal activation of osteoclasts leads to increased bone resorption and insufficient production of osteoblasts leads to impaired bone formation. Osteoclast/osteoblast imbalance underlies bone loss in RA, including bone erosion, periarticular bone loss and systemic osteoporosis. RANKL is an important mediator of osteoclast production and the key role of osteoclasts in bone erosion has been demonstrated in basic research and the clinical efficacy of antibodies targeting RANKL (148).

It was found that TRPV1 was expressed in chondrocytes (149). TRPV1 channel activation enhances RANKL-mediated differentiation of bone marrow-derived macrophages (BMM) to osteoblasts. TRPV1 channel inhibition reduces RANKL-mediated osteoclast formation. This suggests that although TRPV1 activation by itself does not induce osteoclastogenesis, it has a critical synergistic effect on RANKL-mediated signalling events. *In vivo*, experiments showed the same results, with mice in the TRPV1 agonist (curcumin or CAP) group having a higher degree of trabecular osteoclast formation, trabecular microstructure erosion, bone loss and reduced vertebral bone density, comparable to the ovariectomized group (150). Capsazepine, a TRPV1 ion channel antagonist, inhibits osteoclast bone resorption and prevents ovariectomy-induced bone loss in mice (151). The reduced osteoclast formation in TRPV1 KO mice is a strong indication of the important role of TRPV1 in bone destruction. *In vitro*, experiments have further explored the mechanism of action of the TRPV1 channel in regulating bone destruction. In bone marrow-osteoblast co-cultures and RANKL-generated osteoblast cultures, capsazepine inhibited osteoclast formation and bone resorption in a dose-dependent manner. The TRPV1 agonist CAP enhances RANKL-stimulated osteoclast formation. CAP also inhibits RANKL-induced phosphorylation of IkappaB and ERK1/2 and causes apoptosis in mature osteoblasts, and inhibits alkaline phosphatase activity and bone nodule formation in calcified osteoblast cultures (151). The use of glucocorticoids is also known to be an important cause of joint destruction and bone loss in RA. TRPV1 can be induced to dysregulate by glucocorticoids and promote osteoclastogenesis. The pharmacology of TRPV1 significantly inhibited the over-activation of osteoclasts, suggesting a therapeutic use of this channel in protecting against glucocorticoid-induced bone loss (152).

In summary, the TRPV1 channel regulates osteoclast activation and apoptosis and also affects osteoblasts, playing an important role in RA articular bone/chondral destruction. Modulation of the TRPV1 channel may be a promising therapeutic idea to mitigate bone destruction in RA.

## 4 TRPV1-targeted therapy

**Figure 2:**
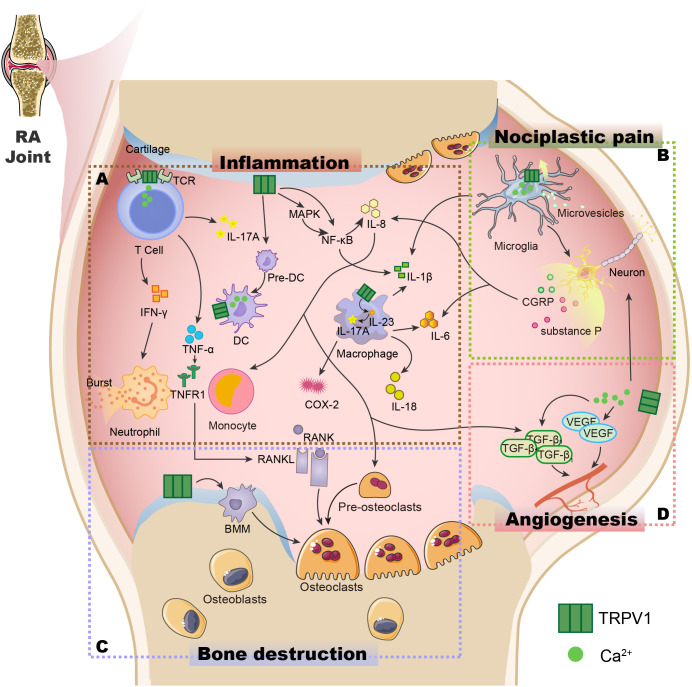
The potential role of the TRPV1 channel in the pathogenesis of RA. **(A)** TRPV1 is expressed not just in synovial cells but also in immune cells such as T cells (44), macrophages (47), and dendritic cells (68), controlling immune cell activity, influencing cytokine release, and causing inflammation in the body. **(B)** The opening of the TRPV1 channel induces increased Ca^2+^ influx, activates microglia (131), promotes the release of the neuropeptide substance P and CGRP from sensory nerve fibres (116, 126), causes neuron sensitization and the release of cytokines, and results in nociplastic pain. **(C)** TRPV1 is expressed in chondrocytes, induces RANK/RANKL production, promotes the maturation of pre-osteoclasts, and mediates the formation of osteoclasts from BMM, leading to bone destruction (150, 151). **(D)** The opening of the TRPV1 channel can cause an increase in Ca^2+^ influx, promoting VEGF and TGF-β1 generation (144), and inducing angiogenesis. TCR, T-cell receptors; IFN-γ, Interferon-γ; IL-17A, Interleukin-17A; Pre-DC, Immature dendritic cells; DC, Dendritic cells; TNF-α, Tumor Necrosis Factor-α; TNFR1, Tumor necrosis factor receptor 1; MAPK, Mitogen-activated protein kinase; NF-κB, Nuclear factor kappa-B; IL-8, Interleukin-8; IL-1β, Interleukin-1β; IL-23, Interleukin-23; COX-2, Cyclooxygenase-2; RANK, receptor activator of NF-κB; RANKL, Receptor activator of NF-κB ligand; CGRP: Calcitonin gene-related peptide; VEGF, Vascular endothelial growth factor; TGF-β1,Transforming growth factor-β1.

The above studies shed light on the key role of TRPV1 in the mechanisms of inflammation and pain in RA(**Figure 2**). In recent years, TRPV1-targeted drugs have received much attention in inflammatory arthritis, e.g. RA, OA, gouty arthritis, etc. Data on the modulation of the TRPV1 channel for arthritis are summarised in **Table 1**. Many clinical trials are also being conducted to treat arthritis and pain by targeting TRPV1(**Table 2**).

**Table 1:** Summary of studies modulating TRPV1 in the treatment of inflammatory arthritis.

Drugs	Dosage	Disease models	Medication method	Drug type	Results	Citation
CAP	5、10μM	OA	i.a.	TRPV1 agonist	Increase mechanically evoked responses	(13)
	0.05μg	RA	i.d.	TRPV1 agonist	Induce vasodilatation in the skin overlying joints	(153)
RTX	30-70-100μg/kg (pretreatment, 3 days)	RA	i.h.	TRPV1 agonist	Increased joint oedema; Attenuate late mechanical hyperalgesia	(154)
	10µg/time	OA	Intraarticular injection	TRPV1 agonist	Suppress pain, improve gait and weight bearing	(155)
Oleylethanola-mine	10−11M to 10−6M	RA		TRPV1 agonist	Combination with the COX-2 inhibitor nimesulide significantly reduced cytokine and MMP-3 production	(156)
Palmitoylethan-olamine	10−11M to 10−6M	RA		TRPV1 agonist	Alone significantly reduced IL-6 and IL-8 secretion by RASF	(156)
Anandamide	10−6M/10−8M	RA		TRPV1 agonist	Reduces IL-6, IL-8 and TNF production by primary mixed synoviocytes	(156)
	860nmol	RA		TRPV1 receptor agonist	Induce excitability of notice price afferent subpopulations and pain	(157)
SA13353	10mg/kg	RA	p.o.	TRPV1 agonist	Inhibit TNF-α production; reduce the hind paw swelling and joint destruction	(158)
SB-366791	10nmol	Gout	Inject/Paw	TRPV1 selective antagonist	Reduce Persistent pain sensation and oedema	(159)
	0.1nmol	Gout	Inject/Paw	TRPV1 selective antagonist	No effect	(159)
A-889425	10-300μmol/kg	OA	p.o.	TRPV1 receptor antagonist	Alleviated grip force impairment	(12)
	10、30μmol/kg	OA	i.v.	TRPV1 receptor antagonist	Reduced the responses of nociceptive specific neurons	(12)
JNJ-17203212	0.075、0.15 mg/100μL	OA	i.a.	TRPV1 antagonist	Significantly attenuated weight-bearing asymmetry; inhibits mechanically evoked responses of knee joint afferents	(13)
Capsazepine	600ng	TMD	Inject/TMJ	TRPV1 antagonist	Significantly attenuated allodynia of the inflamed TMJ induced by intra-TMJ injection of CFA	(160)
	1mg kg -1	RA		TRPV1 antagonist	Produce anti-hyperalgesia and anti-nociception	(157)
AMG9810	30pmol	Gout	Inject/Paw	TRPV1 receptor antagonist	Largely prevented nociceptive and edematogenic responses to MSU	(161)
ABT-116	10mg/kg	Synovitis	Intraarticular injection	TRPV1 proprietary antagonist	Attenuate Synovitis and lameness	(162)
Eucalyptol	600 mg·kg -1	Gout	i.p.	Downregulate the expression of TRPV1	Attenuate mechanical allodynia and ankle oedema	(163)
OMDM-198	1mg/kg	OA	i.p.	TRPV1 antagonist	Significant antinociceptive effects	(164)
AMG9810	30mg/kg	OA	i.p.	TRPV1 receptor antagonist	Reverse thermal hyperalgesia and block Pain	(165)
APHC3	0.1mg/kg	RA	i.h.	Mode-selective TRPV1 antagonist	Reverse pain-induced paw dysfunction	(166)
A-995662	100 mmol/kg	OA	p.o.	TRPV1 antagonist	Reduce spinal glutamate and CGRP release; analgesic efficacy in pain	(167)
Cannabidiol	10µM/20µM	RA		TRPV1 agonist	Reduce cell viability and proliferation of RASF	(168)
SZV1287	20mg	RA	i.p.	TRPV1 antagonist	Decrease hyperalgesia, L4-L6 spinal dorsal horn microgliosis, oedema and myeloperoxidase activity	(169)
SAFit2	10mg/kg	Neuropathic pain	i.p.	Desensitizes the TRPV1	Diminish excessive neuroinflammation and central sensitization	(117)
Fish oil concentrate		Pain induced by heat	p.o.	Reduce expression of TRPV1	Significantly reduces sensitivity to heat-induced pain	(170)
ShexiangZhuifeng Analgesic Plaster		RA	External use	Downregulate the expression of TRPV1	Significantly ameliorated arthritis scores and paw thickness; improve pathological damage of synovial joints; remarkably alleviated pain in CIA rats	(171)

**Table 2:** Drugs targeting TRPV1 channel in clinical development.

Action	Drug	Company	Therapy Area	Highest development status	ClinicalTrials.gov identifier	Citation
TRPV1 agonist	Capsaicin	Not Assigned	Pain	Launched	N/A	
TRPV1 agonist	Zucapsaicin	Sanofi-Aventis Canada Inc	Arthritis	Registered	N/A	
TRPV1 antagonist	JNJ-39439335	Johnson&Johnson Pharmaceutical Research&Development, L.L.C.	Arthritis	Phase 1	NCT00933582	
TRPV1 antagonist	JNJ-39439335	Johnson&Johnson Pharmaceutical Research&Development, L.L.C.	Pain	Phase 1	NCT01006304	
TRPV1 antagonist	NEO6860	Neomed Institute	Arthritis	Phase 1	NCT02337543	
TRPV1 agonist	CNTX-4975	Centrexion Therapeutics Corp	Arthritis	Phase IIb	NCT02558439	(172)
TRPV1 antagonist	V116517	Purdue Pharma	Pain and sensitization	Phase 1	N/A	(173)
TRPV1 antagonist	AZ12048189	AstraZeneca	Local tissue inflammation and pain	Phase 1	N/A	(174)
TRPV1 antagonist	SB-705498	Addenbrooke’s Centre for Clinical Investigation	Heat-evoked pain	Phase 1	N/A	(175)
TRPV1 antagonist	AMG517	Amgen Inc	Pain	Phase 1	N/A	(176)

It is clear from the above that TRPV1 is a highly druggable target. The development of TRPV1-targeted drugs for arthritis is actively pursued worldwide. There is considerable evidence in the table that TRPV1 channel inhibition plays an important role in reducing joint oedema and destruction, as well as relieving inflammation and pain. TRPV1 channel agonists can also target intractable pain and chronic pain after inflammatory remission through desensitization, suggesting that targeting TRPV1 has great potential for the treatment of RA. However, the development of TRPV1-targeted drugs has not always been smooth. TRPV1 has complex regulatory functions and is essential for the maintenance of normal body temperature (117). Systemic antagonism of TRPV1 can damage harmful heat sensations in human skin, increasing the harmful heat threshold and leading to accidents such as burns and scalds (177). Antagonizing TRPV1 also interferes with body thermoregulation, leading to excessive body temperature rise (172). The use of TRPV1 agonists such as CAP causes a strong initial pain response and induces vasodilation and the desensitisation and toxicity doses are relatively close to each other, making adverse effects difficult to control (178).

Although these issues have lowered expectations, there has been some promising progress. Region-specific antagonism of TRPV1 can exert analgesic effects without causing hyperthermia (179). “Non-stimulatory” TRPV1 agonists have been developed, such as Olvanil (NE19550) and MRD-652, both of which have shown promise in inflammatory pain models (178, 180), its clinical value, as yet, remains to be proven. In addition, we can also look for natural products that have a regulatory effect on the TRPV1 channel. Given the complexity of TRPV1 function, keeping TRPV1 activity within the physiological range and reducing its sensitizing effects that occur in pathophysiological pain states may be a more promising research and development Strategy.

## 5 Conclusion

The therapeutic role of TRPV1 is a topic that cannot be ignored in the field of inflammation and pain, with some scholars even calling it the “holy grail of pain management” (181). Based on the evidence reviewed, it is clear that TRPV1 plays a central role in the pathology of RA. Excessive activation of TRPV1 leads to immune cell dysfunction and excessive release of inflammatory factors that mediate inflammation in the body. Activation of the TRPV1 channel mediated by neuropeptide release and microglia activation induces nociplastic pain after inflammation control. TRPV1 also plays an important role in angiogenesis and cartilage destruction. Pain and inflammation, angiogenesis, and cartilage destruction are all important parts of the treatment of RA. Thus, TRPV1 is a remarkably promising target for RA therapy, especially in pain management. This is because interfering with its activity alters the function of multiple signalling pathways in the pathogenesis of RA, thereby slowing its progression. Research on TRPV1 channel-targeting drugs is complex and demanding, and a better understanding of TRPV1 function and post-antagonism in the RA paradigm should accelerate the development of TRPV1-targeted modulators.

## Author contributions

This article is mainly written by YQ. YF and YL wrote part of the manuscript and proofread the manuscript. CL, BX, and QZ helped us collect literature information and draw pictures. PJ reviewed the manuscript and proposed final revisions. All authors contributed to the article and approved the submitted version.
